# A lift in snail's gut provides an efficient colonization route for tardigrades

**DOI:** 10.1002/ecy.3702

**Published:** 2022-04-29

**Authors:** Tommi Vuori, Sara Calhim, Matteo Vecchi

**Affiliations:** ^1^ Department of Biological and Environmental Science University of Jyvaskyla Jyvaskyla Finland

**Keywords:** Arianta, dispersal, gastropodochory, Helicidae, phoresis, Tardigrada, zoochory

Colonization dynamics of microscopic invertebrates and the relative importance of different dispersal vectors are largely unknown. Although wind and water are generally assumed to be the dominant dispersal mechanisms for meiofauna, animal‐mediated dispersal has received relatively little attention (Fontaneto, 2019). A few observational studies suggest the capability of tardigrades to colonize new habitats via ingestion (endozoochory) by gastropods and birds (Fox & García‐Moll, 1962; Robertson et al., 2020), but direct evidence is lacking. An astonishing diversity of organisms has been found to survive the passage through the gut of terrestrial gastropods, both from field surveys and experimental studies: lichen (Boch et al., 2011), moss and fern spores (Boch et al., 2013), plant seeds (Türke et al., 2010), protozoans, rotifers, nematodes, collembolans, and oribatid mites (Türke et al., 2018). Gastropods can disperse whole micro‐ecosystems and have a deep influence on the genetic and spatial structure of microinvertebrate populations (Türke et al., 2018). Here we report both observational and experimental evidence for the ability of tardigrades to survive, and later reproduce, after a passage through the gastrointestinal tract of the terrestrial gastropod *Arianta arbustorum* (Linnaeus 1758).

We recovered active tardigrades from two genera (*Macrobiotus* and *Hypsibius*) in 25% of the feces from wild *A. arbustorum* (*n* = 7/28 individuals, Figure 1c), confirming an earlier report by Fox and García‐Moll (1962). A total of 10 tardigrades were recovered from wild snail feces of which 5 were alive. This proportion (50.0%) is not statistically different from the proportion of live mites recovered by Türke et al. (2018) (69.9% [*n* = 120/172]; Fisher Exact probability test *p* = 0.337). In addition, two thriving cultures of tardigrades (*Hypsibius* cf. *allisoni*) were obtained from individuals collected from the snail feces. To our knowledge, this is the first record of tardigrades’ ability to reproduce after passing through an animal's gut. Tardigrades of the species *Macrobiotus ripperi* Stec, Vecchi & Michalczyk, 2021 were artificially fed to *A. arbustorum* snails in a laboratory experiment to quantitatively assess (1) the survival of ingested tardigrades, and (2) the time spent in the snail's gut. Overall, 31.4% [*n* = 218/694] of the ingested tardigrades were defecated alive. Although this survival is statistically different and about half of what was observed for oribatid mites by Türke et al. (2018) (58% [*n* = 40/69]; Fisher Exact probability test *p* > 0.001), it still shows that tardigrades have the potential to survive snail gut passage, and this could allow their dispersal through endozoochory. In addition to 218 tardigrades defecated alive, 78 dead tardigrades were recovered from the feces of the snails in the laboratory experiment. The remaining 398 individuals not recovered are supposed to have been digested and completely destroyed by the snail's digestive system, thus this proportion does not represent the survival success of tardigrades in passing through the gut of snails but can still be compared to the same statistic as for the wild snails as, in that case, the number of ingested tardigrades is unknown. The proportion of alive tardigrades among the ones recovered in the feces did not significantly differ between the wild snails and the snails from the experiment (wild snails 50.0% [*n* = 5/10], experiment snails 73.6% [*n* = 218/296]; Fisher Exact probability test *p* = 0.196). Tardigrades were not defecated uniformly after the ingestion (Figure 1d): 1 day after ingestion, on average only 4.2% of the ingested tardigrades were defecated alive, whereas the peak of 18.4% was reached at 2 days after ingestion. Those defecation rates then declined on the third (0.6%) and fourth (<0.1%) day. The peak of alive tardigrades excreted on day 2 after ingestion could be caused by the retention time of the gut of *A. arbustorum* and/or by an increased probability of digested tardigrades spending more than 2 days in the snail's gut. However, at the moment, it cannot be determined if and to what extent those mechanisms explain the observed results.

**FIGURE 1:**
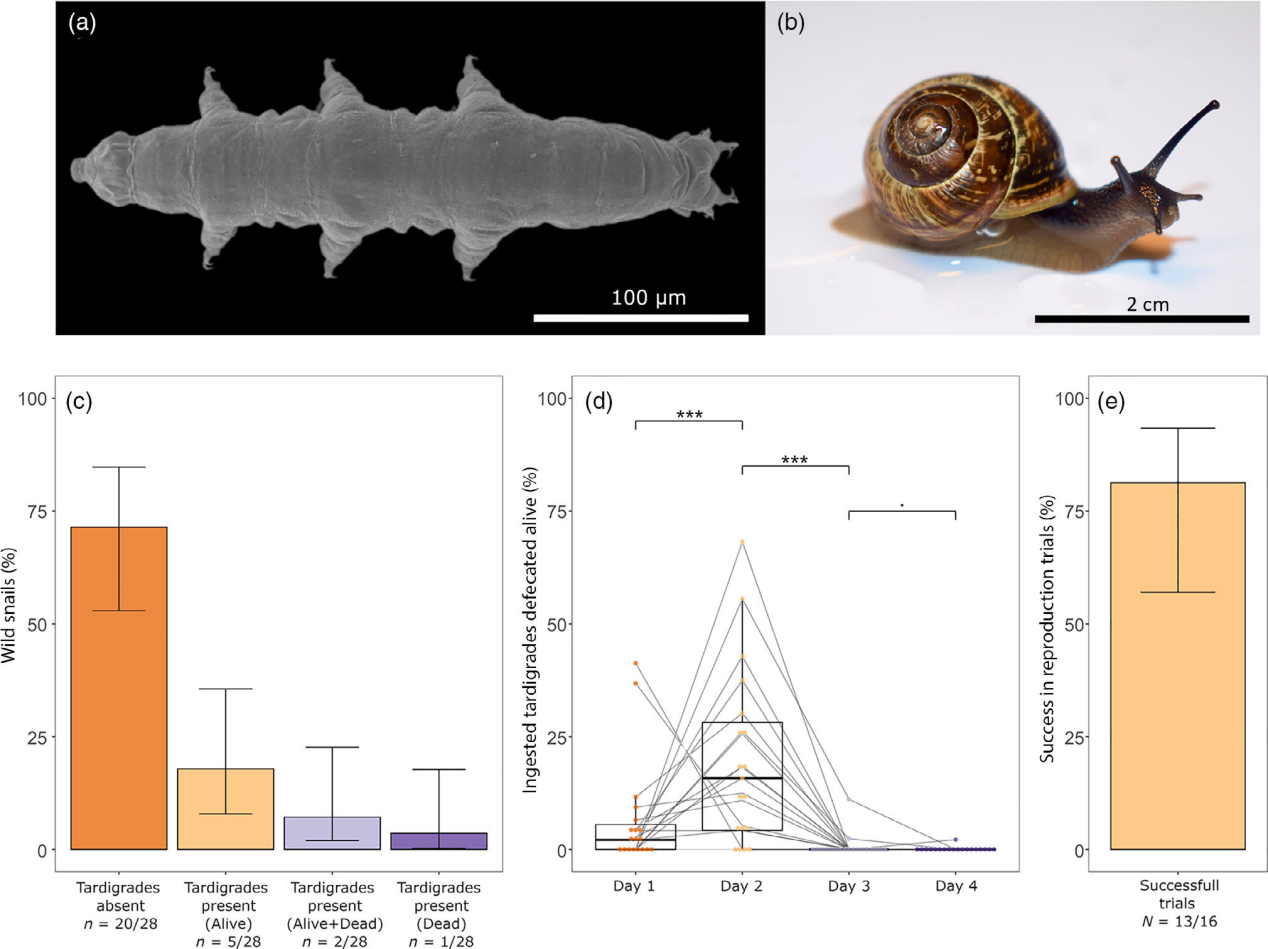
Survival of tardigrades after snail gut passage. (a) *Macrobiotus ripperi*. (b) *Arianta arbustorum*. (c) Percentage of wild snail with and without tardigrades in their feces. Whiskers indicate 95% CI. In 25% of the snails, live tardigrades were recovered from their feces. (d) Percentage of the total ingested *M. ripperi* recovered alive from *A. arbustorum* feces collected at days 1 to 4 post‐ingestion. Gray lines connect the time points of each snail individual. The percentage of alive animals defecated peaked on the second day after ingestion. (e) Percentage of successful *M. ripperi* culturing trials from *A. arbustorum* feces. Whiskers indicate 95% CI. In more than 75% of the trials, populations of tardigrades developed from animals expelled with feces

According to different studies, *A. arbustorum* can move on average 0.18–0.58 m/day with a maximum distance of 4.44–5.48 m/day (Kramarenko, 2014). Accordingly, most of the tardigrades would be dispersed on the second day post‐ingestion that is, on average 1 m (maximum 10 m) away from their original location. On a smooth, wet, two‐dimensional surface in laboratory conditions, tardigrades were reported to move at speeds between 1.98 and 15.81 mm/min (Li & Wang, 2005; Nirody et al., 2021). Hence, theoretically, at directed higher speed movement, tardigrades could match, or even exceed, the distance traveled by a snail (e.g., 48 h at 15.81 mm/min = 45.5 m). However, this scenario is not plausible because, to actively migrate from one point to another, tardigrades must move along surfaces where water film is present: a dry patch of just a few square millimeters becomes an insurmountable dispersal barrier, not to mention the three‐dimensional nature of a natural environment.

Last, we investigated if the tardigrades defecated alive can reproduce (i.e., establish a new population). From the laboratory experiment, all the feces containing live tardigrades from a given snail were pooled and kept in plastic Petri dishes and checked periodically for up to 2 months for the presence of eggs and newborns. These dishes were kept in the same conditions as the laboratory culture (Appendix S1). In most of the Petri dishes (81%, *n* = 13/16, Figure 1e), new and abundant populations were found.

How common and/or important gastropodochory is for tardigrade dispersal remains unclear. Compared to wind dispersal, gastropod‐mediated dispersal may be effective over very small geographic ranges (1–10 m), but it is also more targeted (Boch et al., 2011, 2013), since terrestrial gastropods actively move between humid habitats, where tardigrades are present or can persist. In addition, the deposition of whole ecological communities and nutrients in gastropod feces (Boch et al., 2013; Türke et al., 2018) may promote colonization success.

The high dispersal potential of tardigrades, nematodes, and rotifers is typically attributed to their dormant propagules, and particularly their ability to enter anhydrobiosis, a reversible ametabolic state that allows them to survive almost complete desiccation (Schill, 2019). When in this dry state, they can survive months or years without food and water, resist extreme heat and cold, and be displaced and dispersed by wind (Fontaneto, 2019; Schill, 2019). Since the gut environment is constantly hydrated, it precludes the option of undergoing anhydrobiosis to survive passage through it. Concordantly, we did not detect any signs of anhydrobiosis or other types of dormancies in our experiment.

We propose three possible concurrent mechanisms for our observed survival: first, tardigrades may be too small to be damaged by the buccal mass of *A. arbustorum*; second, the environment inside the digestive system of *A. arbustorum* may simply not be harsh enough to damage tardigrades (see Charrier & Brune, 2003 for gut microenvironments of some helicids), which are shown to tolerate several environmental extremes (Schill, 2019); third, moss or soil particles that are co‐ingested with tardigrades may provide a mechanical protective effect.

In summary, we found that gastropods are not only a viable animal vector for tardigrade dispersal but could also improve the establishment of new populations thanks to the resources their feces provide and the targeted deposition (cf. the stochastic wind vector) in tardigrade‐suitable habitats. Our study highlights the importance of basic ecological research to our currently limited understanding of microorganism dispersal and its links with extreme environment adaptations.

## CONFLICT OF INTEREST

The authors declare no conflict of interest.

## AUTHOR CONTRIBUTIONS

Tommi Vuori collected field samples. Matteo Vecchi and Tommi Vuori designed and conducted the experiment. Matteo Vecchi performed statistical analysis. All authors contributed to interpreting results and writing the manuscript.

## Supporting information


Appendix S1

## Data Availability

Data and code (Vecchi, 2021) are available in Zenodo at https://doi.org/10.5281/zenodo.5584556.
