# Supplementary material for: A lift in snail's gut provides an efficient colonization route for tardigrades
Source: Ecology. 2022 Apr 29;103(7):e3702. doi: 10.1002/ecy.3702 (PMC9285705; doi:10.1002/ecy.3702)
Supplement: Supplementary file 1 — Appendix S1 [file ECY-103-0-s001.pdf]

Supporting information: Vuori, T., Calhim, S. & Vecchi, M. A lift in snail's gut provides an efficient colonization route for tardigrades. *Ecology.*

Appendix S1

Materials & Methods

Wild A. arbustorum collection, feces sampling and husbandry

Twenty-eight *Arianta arbustorum* Linnaeus 1758 individuals were collected from leaf litter in the vicinity of allotment garden site in Jyväskylä, Central Finland (See Table S1). To avoid contaminations, snails were two times carefully washed under running water to get rid of possible soil and moss particles where tardigrades could be present. Feces sampling took place in plastic jars at room temperature (21°C) and snails were provided with small pieces of raw potatoes and kale to detect when the natural gut content (from food ingested before sampling) was defecated based on feces colour (brown for natural feces, whitish/bright green for feces derived from laboratory diet). Snails' excrements were collected twice a day until potato or kale was detected, stored at room temperature in microcentrifuge tube filled with mineral water for up to 5 days, and examined for the presence of tardigrades under a dissecting microscope (snails A1 and A3-A22) or placed on a petri dish immediately after collection and soaked in mineral water until emergence of alive tardigrades (snails B1-B3 and B6-B9). Tardigrades were classified as alive or dead based on motility and eventual decomposition signs of the carcasses. For long term husbandry and artificial tardigrade feeding experiment, snails were kept in individual cages lined with paper towels in a climatic chamber at 16C with a 12:12 light:dark photoperiod, nebulized with tap water twice a week and fed *ad libitum* with moistened fish food flakes (TetraMin Tropical Flakes, Tetra).

Table S1. Sampling locations of wild *Arianta arbustorum*.

**Description**	**Coordinates (WGS84)**	**Number of snails (Snails IDs)**	
		**September sampling**	**October sampling**
Interface of cultivated field and deciduous forest. Dominant tree species were *Prunus padus*, *Alnus incana* and *Salix* sp. Forest floor was covered with dead wood and leaf litter.	62.24859 25.72324	4 (A1, A3-A5)	
Edge of deciduous forest site. The dominant tree species were *Betula pendula* and *Salix* sp. Herbs and shrubs layer dominated by *Rubus idaeus* and *Epilobium angustifolium*. Forest floor covered by leaf litter and moss patches.	62.24897 25.72657	4 (A6-A9)	7 (B1-B3, B6-B9)
A small pile of dead wood on a land strip between allotment gardens. Vegetation around the pile was dominated by *Urtica dioica* and *Elytrigia repens*. Some other unidentified grass species were present. Close to the pile some very small patches of moss were present.	62.24878 25.72725	13 (A10-A22)	

Experimental feeding

Snails were kept in captivity for at least 1 month to ensure that no tardigrade was left in their gut from the wild. Snails were starved for ~50 hours and then fed with a wet mixture of cornstarch and Hylocomiaceae moss (1:20 w:w of cornstarch and wet moss) grinded using scissors and defaunated by boiling for 15 minutes. To the cornstarch/moss mix given to each snail about 50 alive individuals of a laboratory strain of the tardigrade *Macrobiotus ripperi* Stec, Vecchi & Michalczyk, 2021 were added. The tardigrade laboratory strain was started from individuals collected in Jyväskylä (WGS84: 62.223508, 25.772324) cultured in plastic petri dishes (5 cm diameter) at 16C with 2:22 light:dark photoperiod and fed with algae (*Chlorella* sp. and *Chlorococcum hypnosporum* from Sciento Ltd, UK). The cornstarch/moss mixture was found to be palatable by snails and not be deleterious for tardigrades in preliminary tests. Snails were allowed to feed on the mixture for 24 hours, after which, the uneaten food

was inspected to count the uneaten tardigrades. To facilitate feces inspection snails were then fed with cornstarch paste or wet chromatographic paper with small number of fish flakes after the feeding trial. Snails were fed with tardigrades after starvation (day 0), then the feces were collected daily for 4 days and inspected for tardigrades. The experiment was stopped at day 4, as no more tardigrades were expelled with the snail's feces after.

Reproduction trials

All feces recovered from each snail in the feeding experiment that contained alive tardigrades were pooled by snail id and kept in observation for 8 weeks in a petri dish (5 cm diameter) with mineral water and algae and rotifers as food, at 16C with a 12:12 hours photoperiod. Reproduction was considered to have taken place if eggs and newborns were found during the observation period.

Statistical analysis

We compared the proportion of tardigrades defecated alive over the ingested tardigrades between consecutive days with a generalized linear mixed model (Binomial family, Logit link) using the snail ID as random effect. Data analysis and plotting was performed in R v 4.0.2 (R Core Team 2021) with the packages "lme4" v 1.1-25 (Bates et al. 2015) and "ggplot2" v 3.3.3 (Wickham 2016). Raw data and analysis R script are available at https://doi.org/10.5281/zenodo.5584555.

Literature cited

Wickham, H. 2016. ggplot2: Elegant Graphics for Data Analysis. Springer-Verlag New York.

Bates, D., Maechler, M., Bolker, B., Walker, S. 2015. Fitting Linear Mixed-Effects Models Using lme4. Journal of Statistical Software, 67, 1-48.

R Core Team 2021. R: A language and environment for statistical computing. R Foundation for Statistical Computing, Vienna, Austria. URL https://www.R-project.org/.
